# Construction and validation of a risk prediction model for intraoperative hypothermia in elderly patients undergoing total hip arthroplasty

**DOI:** 10.1007/s40520-023-02500-0

**Published:** 2023-07-25

**Authors:** Bin zhao, Zhe zhu, Wenwen Qi, Qiuli Liu, Qi Zhang, Liping Jiang, Chenglong Wang, Xiaojian Weng

**Affiliations:** 1 Department of Anesthesiology and SICU, School of Medicine, Xinhua Hospital, Shanghai Jiao Tong University, Kongjiang Road 1665, Shanghai, 200092 China; 2 Department of Psychogeriatric, School of Medicine, Shanghai Mental Health Center, Shanghai Jiao Tong University, South Wanping Road 600, Shanghai, 200030 China; 3 Department of Nursing, School of Medicine, Xinhua Hospital, Shanghai Jiao Tong University, Kongjiang Road 1665, Shanghai, 200092 China; 4 Department of Emergency, School of Medicine, Xinhua Hospital, Shanghai Jiao Tong University, Kongjiang Road 1665, Shanghai, 200092 China; 5 Department of Orthopaedic Surgery, School of Medicine, Xinhua Hospital, Shanghai Jiao Tong University, Kongjiang Road 1665, Shanghai, 200092 China

**Keywords:** Intraoperative hypothermia, Risk prediction model, Elderly patients, Total hip arthroplasty

## Abstract

**Aims:**

To construct and validate an intraoperative hypothermia risk prediction model for elderly patients undergoing total hip arthroplasty (THA).

**Methods:**

We collected data from 718 patients undergoing THA in a tertiary hospital from January 2021 to December 2022. Of these patients, 512 were assigned to the modeling group from January 2021 to April 2022, and 206 participants were assigned to the validation group from May 2022 to December 2022. A logistic regression analysis was performed to construct the model. The area under the curve (AUC) was used to test the model’s predictive ability.

**Results:**

The incidence rate of intraoperative hypothermia was 51.67%. The risk factors entered into the risk prediction model were age, preoperative hemoglobin level, intraoperative blood loss, postoperative hemoglobin level, and postoperative systolic blood pressure. The model was constructed as follows: logit (P) = − 10.118 + 0.174 × age + 1.366 × 1 (preoperative hemoglobin level) + 0.555 × 1 (postoperative hemoglobin level) + 0.009 × 1 (intraoperative blood loss) + 0.066 × 1 (postoperative systolic blood pressure). Using the Hosmer–Lemeshow test, the P value was 0.676 (AUC, 0.867). The Youden index, sensitivity, and specificity were 0.602, 0.790, and 0.812, respectively. The incidence rates of intraoperative hypothermia in the modeling and validation groups were 53.15% and 48.06%, respectively. The correct practical application rate was 89.81%. This model had good application potential.

**Conclusions:**

This risk prediction model has good predictive value and can accurately predict the occurrence of intraoperative hypothermia in patients who undergo THA, which provides reliable guidance for clinical work and has good clinical application value.

## Introduction

With the global escalation of the aging process, the incidence of hip fractures in elderly patients has increased significantly [1]. Total hip arthroplasty (THA) is one of the most common surgical treatments for pain relief and physical function recovery. Although its perioperative course is relatively predictable, patients who undergo THA still experience various perioperative complications, especially intraoperative hypothermia (IOH) in geriatric patients.

IOH, defined as a core temperature (CT) < 36.0℃ during surgery, is a common complication among surgical patients [2]. In recent studies, the incidence rate of IOH has ranged from 26.6% to 65% in patients who undergo joint arthroplasty [3–6]. Elderly patients undergoing THA are at high risk of hypothermia during surgery, especially under general anesthesia (GA) [7]. GA and surgery affect the balance between heat production and loss. Previous studies have reported IOH rates of 13.2%–39% after THA [6, 8]. IOH is associated with several adverse effects, including blood loss, surgical site infection, disturbed drug metabolism, postoperative cardiovascular events, prolonged hospital stay, and mortality [9–14]. Despite these effects, IOH remains unresolved. Therefore, future studies should focus on IOH. In this era of personalized medicine, considering that elderly patients do not have the same risk factors for IOH, the development of risk prediction models for IOH is necessary.

A risk prediction model aims to predict the probability of future events, such as whether a patient will develop a disease or not [15]. A systematic review reported an overview of the current eight hypothermia risk prediction models for common surgeries, such as abdominal, thoracic, and gynecological surgeries [16]. In these studies, researchers identified an association between the occurrence of IOH and relevant predictors, such as age, weight, baseline temperature, and fluid volume [17–19]. However, several models failed to show pivotal data, such as sample size considerations, samples with missing data, and model validation, which may lead to a high risk of bias. Additionally, these predictors lead to existing prediction models for IOH that are unsuitable for elderly patients undergoing THA.

Therefore, to enrich IOH prevention for joint arthroplasty surgery, this study aimed to investigate the incidence of IOH, examine the risk factors, and construct and validate a risk prediction model for IOH in elderly patients undergoing THA. The risk prediction model was hypothesized to provide good discrimination and adequate calibration in clinical practice.

## Methods

### Study design and population

This was a prospective cohort study using a convenience sampling method to select patients who underwent THA in a tertiary general hospital with an annual volume of approximately 5000 orthopedic surgeries in Shanghai, China, from January 1, 2021, to December 31, 2022.

The inclusion criteria were as follows: patients (1) aged ≥ 60 years, (2) undergoing elective THA surgery, (3) with preoperative basal CT ranging from 36.5 to 37.5℃, (4) induced with GA, and (5) with good communication or without mental illness.

The exclusion criteria were as follows: patients (1) with premedication that affects the thermoregulatory center, (2) with central hyperthermia, (3) with abnormal thyroid function, (4) with infectious fever, (5) with body temperature regulation dysfunction, (5) who were unsuited for infrared tympanic thermometry, and (6) unwilling to participate in this study.

### Sampling consideration and sample size

Based on the literature review and expert consultations, this study included 42 risk factors for data collection. These risk factors included the following: (1) patient’s general information (age, sex, height, weight, body mass index [BMI], past history [including surgery, anesthesia, smoking, and alcohol histories], comorbidities [including diabetes, hypertension, and cardiovascular and cerebrovascular diseases]); (2) preoperative physiological indicators (preoperative systolic blood pressure, preoperative diastolic blood pressure, preoperative hemoglobin level); (3) relevant surgical information (anesthesia classification, anesthesia time, operating time, awakening time from GA, fluid volume, flushing fluid volume, intraoperative blood transfusion, volume of blood transfusion, intraoperative blood loss, autologous blood transfusion, volume of urine, dosage of opioid drugs, dosage of muscle relaxants, postoperative systolic blood pressure, postoperative diastolic blood pressure, postoperative hemoglobin level); and (4) temperature indicators (preoperative CT, CT at induction of anesthesia, CT after 30 min of GA, CT after 60 min of GA, CT after 90 min of GA, CT after 120 min of GA, minimum intraoperative CT, maximum intraoperative CT, temperature in operating room, temperature of intraoperative fluids, temperature of intraoperative flushing fluids, temperature of intraoperative blood transfusion, CT after tracheal extubation, postoperative CT).

A preliminary experiment with 54 patients in this study showed that the incidence rate of IOH in elderly patients undergoing THA was 64.81% (35/54). The sample size was calculated based on the incidence of IOH in the preliminary experiment. According to the formula, sample size = (events per predictor variable × 5–10) × (1 + [15–20%])/0.6851, 373–778 samples could ensure the validity of the study results. A random seed within the SPSS version 25.0 software was used to draw 70% of the sample size as the modeling group and 30% as the validation group. Ultimately, 545 and 233 samples were included in the modeling and validation groups, respectively.

### Study procedures

To provide reliable results, anesthesiologists and nurse anesthetists were trained in temperature management, temperature measurement, and data collection.

GA was induced with 0.02–0.05 mg/kg midazolam, 1.5–2.0 mg/kg propofol, 0.6 mg/kg rocuronium bromide, and 0.5–1 ug/kg remifentanil and maintained with 4.0–6.0 mg/kg propofol, 5–10 ug/kg remifentanil, 1.5–2 minimum alveolar concentration sevoflurane, and 0.1 mg/kg rocuronium bromide. The bispectral index (BIS) (Narcotrend^®^ Narcotrend-Compact, MT monitorTechnik Gmbh& Co.KG, Germany) has been recommended to guide doses of anesthetics to achieve optimal anesthetic depth. The recommended range of BIS was 40–50 during anesthesia maintenance. Anesthesia induction, intraoperative anesthesia management, and awakening from GA were the primary responsibilities of anesthesiologists.

Operating room temperature was constant at 21–25℃, and room humidity ranged from 30 to 60%. All patients undergoing THA were covered with blankets, sheets, or surgical drapes perioperatively. Room-temperature intravenous fluids, room-temperature flushing fluids, and warm blood products were administered according to routine practice. Intraoperative temperature management was the responsibility of nurse anesthetists.

A noninvasive wireless ear thermometer (BodySTM^®^ temperature transducer, Wismed, Beijing) was used to measure the CT of the participants. Before the induction of anesthesia, a wireless thermometer was inserted into the external auditory canal to measure the tympanic temperature. A previous study has reported that tympanic thermometers have good accuracy and precision for CT measurements [20]. The thermometer was paired with a monitoring module that continuously recorded the tympanic temperature, and the temperature data were uploaded to a terminal server for storage. Tympanic temperature readings were displayed on an electrocardiogram monitor (IntelliVue MX800^®^ patient monitor, PHILIPS, Shanghai) and recorded on a data form by anesthesiologists every 10 min during the operation. The wireless ear thermometer was removed before the participants were discharged from the operating room to postoperative care unit. Intraoperative autologous transfusion (3000P® Blood Recycling machine; JINGJING, Beijing, China) was used to decrease the number of transfusions in patients with large intraoperative blood loss.

### Data collection

This study included 718 elderly patients who underwent THA – 512 participants in the modeling cohort from January 2021 to April 2022 and 206 participants in the validation cohort from May 2022 to December 2022. A data form was developed to collect the demographic data and risk factors for IOH. The data form included the following contents: (1) demographic characteristics (age, sex, height, weight, BMI, past history [including surgery, history, smoking, and alcohol], comorbidities [including diabetes, hypertension, and cardiovascular and cerebrovascular diseases], systolic blood pressure, diastolic blood pressure, hemoglobin level); (2) surgical and anesthetic information (anesthesia classification, anesthesia time, operating time, awakening time from GA, fluid volume, flushing fluid volume, intraoperative blood transfusion, volume of blood transfusion, intraoperative blood loss, autologous blood transfusion, volume of urine, dosage of opioid drugs, dosage of muscle relaxants); and (3) temperature indicators (preoperative CT, CT at induction of anesthesia, CT after 30 min of GA, CT after 60 min of GA, CT after 90 min of GA, CT after 120 min of GA, minimum intraoperative CT, maximum intraoperative CT, temperature in operating room, temperature of intraoperative fluids, temperature of intraoperative flushing fluids, temperature of intraoperative blood transfusion, CT after tracheal extubation, postoperative CT).

The CT was measured before surgery. Nurse anesthetists monitored the CT every 10 min from the induction of anesthesia until the participants left the operating room. Continuous CT data during the operation were obtained using an electrocardiogram. CT < 36℃ was considered IOH. Subsequently, the training nurses obtained demographic data, relevant surgical information, anesthesia information, and physiological indicators from the HIS system of electronic medical records and recorded them in a data form.

### Ethical consideration

This study was approved by the Ethics Committee (No. XHEC-D-2023–005). All patients voluntarily participated in this study and signed an informed consent form.

### Statistical analyses

The SPSS version 25.0 software was used for the statistical analyses. Nomogram was drawn in R version 4.3.1 software to visualized. Regarding statistical description, measurement data following normal distribution are described as means ± standard deviations. Measurement data with non-normal distribution are described as medians and quartiles. Count data are presented as frequencies and percentages. T-tests were used to analyze normally distributed measurement data. The rank-sum test was used to measure data with a non-normal distribution. Chi-squared tests were used to analyze the count data. All risk factors were analyzed using single- and multi-factor logistic analyses. Logistic regression analysis was performed to construct a prediction model. The Hosmer–Lemeshow test and the area under the receiver operating characteristic (ROC) (AUC) were used to verify the prediction model. The practical application efficiency of the prediction model was verified by calculating the sensitivity, specificity, and accuracy of clinical data.

## Results

### Patient selection and flowchart of the study

**Fig. 1:**
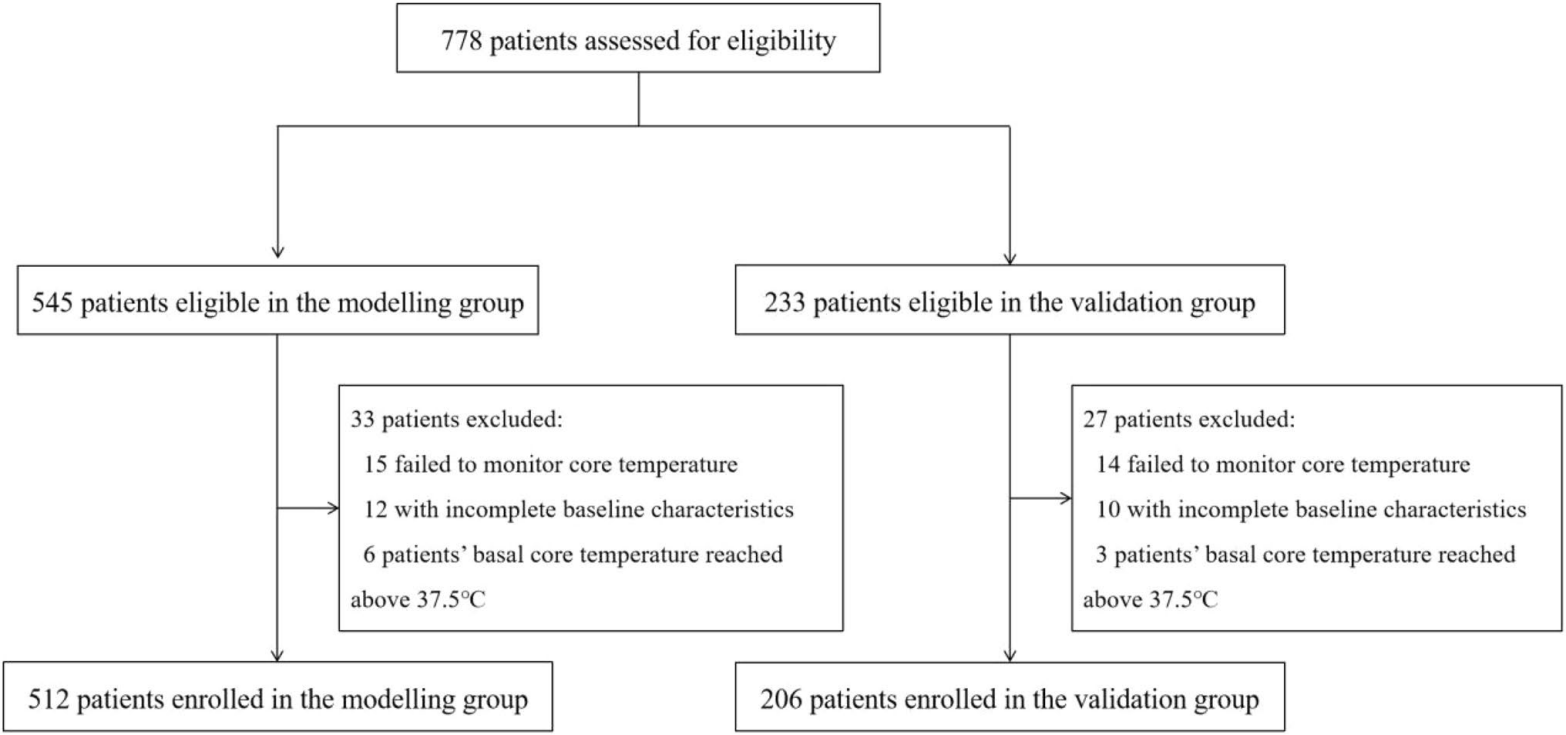
Flowchart of the study

Overall, 778 patients were screened for eligibility, of whom 33 and 27 patients in the modeling and validation groups, respectively, were excluded for the reasons explained in Fig. 1. In total, 718 valid patients were identified, with 512 and 206 in the modeling and validation groups, respectively.

### Demographic characteristic

Among the 718 patients included in the analysis, IOH was observed in 371 patients, with an incidence rate of 51.67%. The 512 patients in the modeling group were divided into the hypothermia and non-hypothermia groups, with 272 patients in the hypothermia group, and the incidence rate of IOH was 53.15%. In the validation group, 99 patients had IOH, with an incidence rate of 48.06%.

In the univariate test, age, BMI, preoperative hemoglobin level, anesthesia time, volume of intraoperative fluids, volume of blood transfusion, intraoperative blood loss, postoperative systolic blood pressure, and postoperative hemoglobin level were significant factors that contributed to IOH (Table 1).

**Table 1:** Demographic characteristic and univariate analysis results in the modeling group (*n* = 512)

	Hypothermia group (*n* = 272)	Non-hypothermia group (*n* = 240)	Statistic value	*P*
Age	75.83 ± 5.650	69.15 ± 5.867	13.105	< 0.001
Sex			0.138	0.890
Male	97 (35.7)	87 (36.3)		
Female	175 (64.3)	153 (63.7)		
Height (cm)	169.30 ± 5.292	169.09 ± 5.628	0.436	0.663
Weight (kg)	63.83 ± 7.501	64.10 ± 7.847	− 0.397	0.691
BMI (kg/m^2^)	21.84 ± 2.69	22.70 ± 2.96	− 3.452	0.001
Past history				
Surgery history	164 (60.3)	137 (57.1)	0.736	0.462
Anesthesia history	164 (60.3)	137 (57.1)	0.736	0.462
Smoking history	28 (10.3)	23 (9.6)	0.267	0.789
Alcohol history	14 (5.1)	17 (7.1)	− 0.916	0.360
Comorbidities				
Diabetes	55 (20.2)	63 (26.3)	− 1.608	0.108
Hypertension	169 (62.1)	140 (58.3)	0.876	0.382
Cardiovascular disease	176 (64.7)	167 (69.6)	− 1.173	0.242
Cerebrovascular disease	121 (44.5)	92 (38.3)	1.411	0.159
Preoperative systolic blood pressure (mmHg)	152.42 ± 18.022	150.91 ± 18.806	0.927	0.354
Preoperative diastolic blood pressure (mmHg)	65.18 ± 6.377	65.80 ± 6.494	− 1.081	0.280
Preoperative hemoglobin level (g/L)	12.3 ± 0.792	13.2 ± 0.977	− 10.666	< 0.001
Anesthesia classification			− 1.377	0.169
II	67 (24.6)	47 (19.6)		
III	205 (75.4)	193 (80.4)		
Anesthesia time (min)	152.31 ± 8.292	150.73 ± 7.034	11.507	< 0.001
Operating time (min)	137.03 ± 7.305	136.44 ± 8.054	0.866	0.387
Awakening time from general anesthesia (min)	9.99 ± 3.015	9.48 ± 2.975	1.937	0.053
Volume of intraoperative fluids (mL)	1738.34 ± 149.154	1769 ± 149.497	− 2.334	0.020
Volume of flushing fluids (mL)	1752.21 ± 141.665	1744.17 ± 104.538	0.723	0.470
Intraoperative blood transfusion			1.134	0.257
Autologous	177 (65.1)	69 (28.7)		
No blood transfusion	95 (34.9)	171 (71.3)		
Volume of blood transfusion (mL)	136.69 ± 119.334	51.15 ± 89.854	9.225	< 0.001
Intraoperative blood loss (mL)	392.81 ± 109.595	288.73 ± 100.376	11.152	< 0.001
Volume of urine (mL)	72.86 ± 9.214	72.88 ± 9.940	− 0.002	0.983
Postoperative opioid drugs			/	/
Hydromorphone	272 (100.0)	240 (100.0)		
Dosage of opioid drugs (mg)	0.698 ± 0.177	0.713 ± 0.170	− 0.955	0.340
Muscle relaxants			/	/
Rocuronium bromide	272 (100.0)	240 (100.0)		
Dosage of muscle relaxants (mg)	39.80 ± 4.730	39.92 ± 4.818	− 0.281	0.779
Postoperative systolic blood pressure (mmHg)	124.82 ± 6.072	123.42 ± 6.015	2.621	0.009
Postoperative diastolic blood pressure (mmHg)	62.17 ± 4.333	62.43 ± 4.323	− 0.678	0.498
Postoperative hemoglobin level (g/L)	10.9 ± 0.840	11.2 ± 1.012	− 4.310	< 0.001
Preoperative core temperature (℃)	36.75 ± 0.90	36.74 ± 0.098	1.862	0.069
Core temperature at induction of anesthesia (℃)	36.75 ± 0.099	36.75 ± 0.093	1.060	0.289
Operating room’ s temperature (℃)	22.24 ± 0.158	22.25 ± 0.156	− 0.752	0.452
Temperature of intraoperative fluids (℃)	23.97 ± 0.576	23.98 ± 0.583	− 0.325	0.745
Temperature of intraoperative flushing fluids (℃)	23.59 ± 0.848	23.45 ± 0.880	1.809	0.071
Temperature of intraoperative blood transfusion (℃)	22.98 ± 0.605	22.96 ± 0.581	0.266	0.790
Postoperative core temperature (℃)	36.4 ± 0.068	36.4 ± 0.068	− 1.855	0.064

### Prediction model of intraoperative hypothermia during total hip arthroplasty

Nine variables with statistical significance were identified as candidate predictors, and only five variables were retained in the model. The results showed that age, preoperative hemoglobin level, postoperative hemoglobin level, intraoperative blood loss, and postoperative systolic blood pressure were associated with IOH (Table 2). In the multivariate model, elderly patients (odds ratio [OR], 1.190; 95% confidence interval [CI], 1.140–1.242; *P* < 0.001) were at a high IOH risk. Patients with lower preoperative (OR, 1.255; 95% CI 1.178–1.365; *P* < 0.001) and postoperative hemoglobin (OR, 1.742; 95% CI 1.225–2.479; *P* = 0.002) levels were more likely to develop IOH. Intraoperative blood loss was associated (OR, 1.009; 95% CI 1.006–1.01; *P* < 0.001) with IOH. Postoperative systolic blood pressure was 1.068 (95% CI 1.024–1.115; *P* = 0.002) higher than the odds of IOH. The model was constructed as follows: logit (*P*) = − 10.118 + 0.174 × age + 1.366 × 1 (preoperative hemoglobin level) + 0.555 × 1 (postoperative hemoglobin level) + 0.009 × 1 (intraoperative blood loss) + 0.066 × 1 (postoperative systolic blood pressure).

**Table 2:** Multivariate regression results of intraoperative hypothermia (n = 512)

Independent	*β* value	Standard error	Wald	*P*	OR	95% CI
Age	0.174	0.022	62.435	< 0.001	1.190	1.140–1.242
Preoperative hemoglobin level	1.366	0.183	55.819	< 0.001	1.255	1.178–0.365
Postoperative hemoglobin level	0.555	0.180	9.521	0.002	1.742	1.225–2.479
Intraoperative blood loss	0.009	0.002	29.510	< 0.001	1.009	1.006–1.01
Postoperative systolic blood pressure	0.066	0.022	9.304	0.002	1.068	1.024–1.115
Constant	–10.118	5.007	4.083	0.043		

### Prediction effect of intraoperative hypothermia risk prediction model after internal verification during total hip arthroplasty

**Fig. 2:**
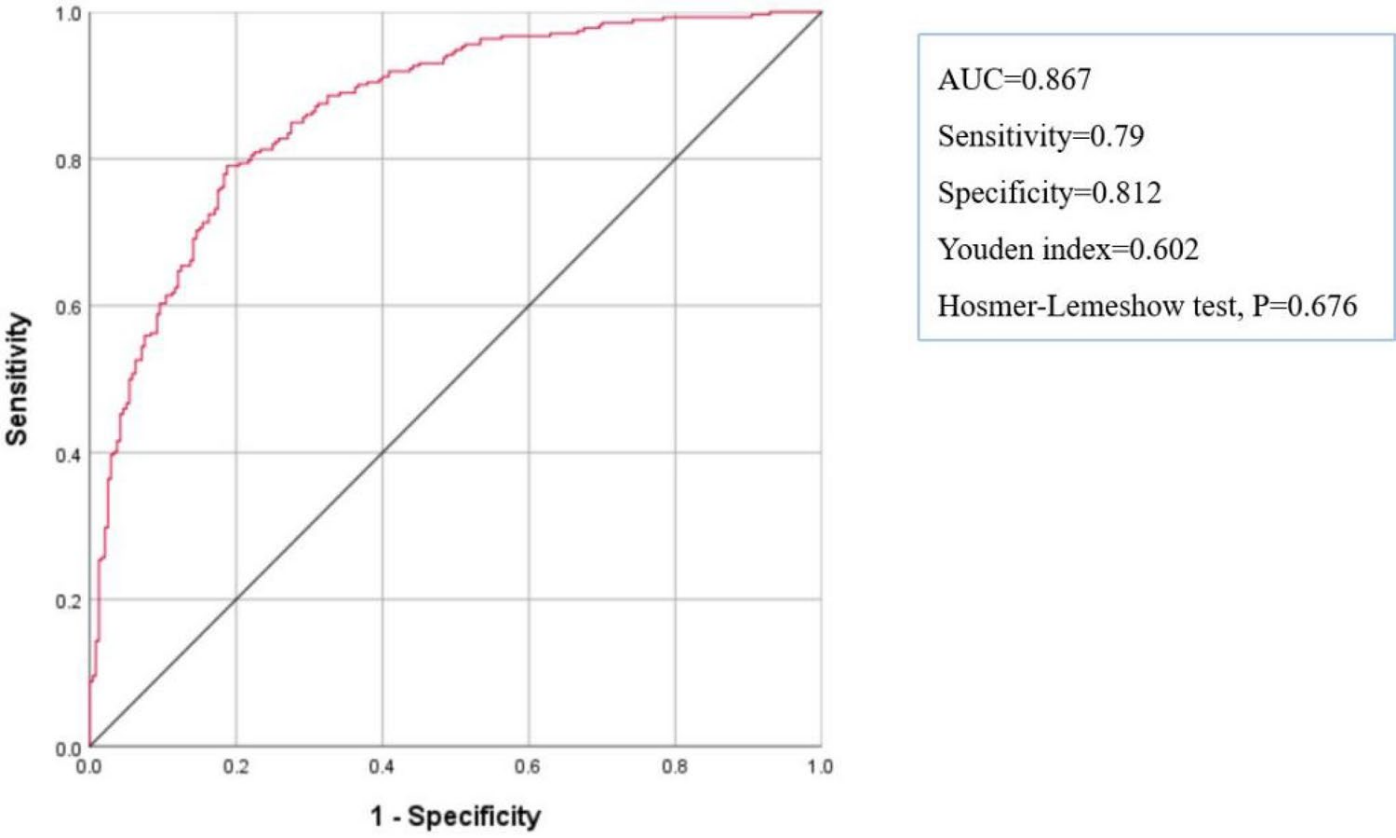
Receiver operating characteristic curve and internal validation data

**Fig. 3:**
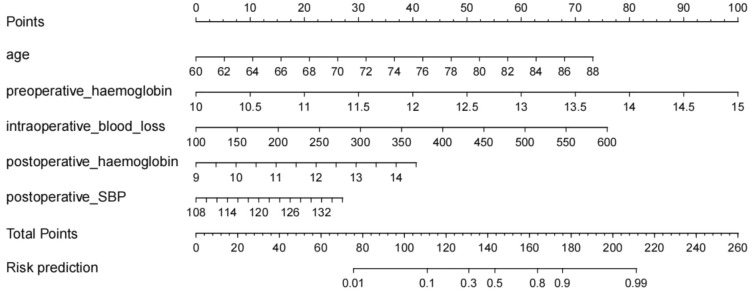
Nomogram

Using the Hosmer–Lemeshow test on the prediction model, we obtained a P value of 0.676, which indicates excellent calibration performance. ROC curves were used to test the sensitivity and specificity of the risk model for predicting IOH. The Youden index is a frequently used summary measure of ROC curves. The maximum Youden index is the best critical value of the prediction model. The AUC, Youden index, sensitivity, and specificity were 0.867, 0.602, 0.79, and 0.812, respectively (Fig. 2). The nomogram is shown in Fig. 3.

### External validation of intraoperative hypothermia risk prediction model during total hip arthroplasty

The external validation of the prediction model was conducted on 206 patients. Ninety-nine patients underwent IOH, with an incidence rate of 48.06%. Of the 102 patients identified by the risk prediction model, hypothermia occurred in 89 patients, with a sensitivity of 89.90%. In total, 107 patients were predicted by the model; 96 patients were successfully predicted, with a specificity of 89.72%. The total accuracy of the model was 89.91% (89 + 96/206) (Table 3).

**Table 3:** Clinical external validation data (*n* = 206)

	Hypothermia	Non-hypothermia
Predicted values	102	104
Actual value	99	107
Correct value	89	96
Error value	11	11
Specificity		89.72%
Sensitivity	89.90%	
Accuracy	89.81%	

## Discussion

IOH is the most common complication of surgery and can increase the risk of unfavorable perioperative events. Recently, most medical staff have recognized the several adverse effects of IOH and have gradually started to use risk prediction models and take early interventions to prevent IOH. Intraoperative prediction models for patients undergoing GA surgery can effectively predict and prevent IOH [21–23]. For example, the IOH risk prediction model developed by Dai et al. demonstrated helpful discrimination and adequate calibration [21]. THA has the characteristics of a large incision and trauma, but uses neuraxial or regional anesthesia, which is different from laparoscopic minimally invasive surgery and GA in patients with IOH. Neuraxial anesthesia impairs central thermoregulatory, reducing vasoconstriction and shivering threshold by 0.5℃ and elevating the sweating threshold by 0.3℃. This combined effect triples the interthreshold range, triggering a physiological response to the cold [24]. In addition, neuraxial anesthesia blocks the efferent nerves that regulate autonomic thermoregulatory defenses and dramatically impairs vasoconstriction and shivering [25]. Shortly after the administration of the neuraxial block, vasodilation shifts warm blood from the core to cooler peripheral tissues, resulting in a decrease in CT and redistribution hypothermia. Because of impaired thermoregulatory control, this drop in temperature may be sustained during anesthesia. In the case of regional anesthesia, redistribution of body heat is caused by centrally mediated vasodilation and direct inhibition of vasoconstriction (and shivering) in the regionally blocked area. Internal core-to-peripheral redistribution of body heat is the most important cause of hypothermia after neuraxial or regional anesthesia. To avoid bias, neuraxial and regional anesthesia were excluded from this study.

Based on a literature review, most joint replacement studies mixed THA and total knee arthroplasty (TKA) to construct risk prediction, which might affect the discrimination of risk factors and predictive values [26, 27]. Moreover, the incidence rate of IOH after TKA was significantly lower than after THA [8]. Therefore, it is necessary to study the risk factors and develop a predictive model for IOH in patients who undergo THA [28].

In this study, we observed an overall incidence rate of IOH of 51.67% in the patients who underwent THA. Older patients with a lower preoperative hemoglobin level before anesthesia, higher postoperative systolic blood pressure, lower postoperative hemoglobin level after surgery, and experienced more blood loss were susceptible to IOH. We proposed a risk prediction model that aimed to identify patients who underwent THA with a high risk of IOH. The Hosmer–Lemeshow test showed a *P* value of 0.676 (AUC, 0.867 [95% CI 0.836–0.898]), and the prediction success rate reached 89.81% in actual detection, confirming that this model has a good discriminative ability for prediction. Therefore, this model could be used to predict high risk factors for IOH in elderly patients who underwent THA and provide a reference for preventive interventions.

The mean age of patients in the IOH group was 75.83 ± 5.560 years, which had a significantly higher age than the non-hypothermia group (*P* < 0.001). Patients who undergo GA have an increased risk of IOH with advanced age [29–31]. The association between age and IOH may be due to a decrease in the thermoregulatory vasoconstriction threshold in patients under general anesthesia with age [18, 32]. Geriatric patients have a low metabolic rate, poor thermoregulatory ability, and high susceptibility to IOH.

IOH was more likely to occur at lower BMI (*P* = 0.001). In a previous study, being overweight was protective owing to the body fat maintaining heat balance by triggering vasoconstriction early when the CT decreased in obese patients [33]. Another study reported a strong positive association between serum leptin levels and fat [34]. Leptin stimulates energy consumption in brown adipose tissue and increases metabolic rate and body temperature. Therefore, the lower the BMI, the less leptin is secreted and the higher the likelihood of hypothermia.

A significant finding of this study was that univariate analysis showed that anesthesia time, volume of intraoperative fluids, and volume of blood transfusion were important factors influencing IOH. These results are consistent with those of a previous study, which reported that a longer duration of anesthesia (> 2 h) increased the risk of hypothermia [35]. In the present study, the administration of a lower volume of intraoperative fluid was significantly associated with IOH. However, this finding was inconsistent with those of previous studies [36, 37]. These variations could be due to differences in the technique of measurement and may be due to difference in sample population. From these results, clearly, IOH is associated with the volume of blood transfused during THA. Schmied et al. also found that intraoperative hypothermia increased blood transfusion requirements in patients undergoing THA [38, 39].

Compared with preoperative hemoglobin level before surgery, the mean hemoglobin level in the hypothermia group was significantly lower than that in the non-hypothermia group (*P* < 0.001), similar to the postoperative hemoglobin levels (*P* = 0.002). We found that greater intraoperative blood loss was an important factor in IOH development. A reasonable explanation is that with increasing intraoperative blood loss, hemoglobin level also decreases. IOH also reduces the adhesion ability of platelets and subsequent coagulation processes [40]. In this study, IOH was independently associated with lower hemoglobin levels and blood loss after THA. A previous study reported that maintaining body temperature during spinal surgery under GA could reduce blood loss and transfusion rates. However, the relationship among IOH, hemoglobin level, and blood loss in patients undergoing orthopedic surgery remains controversial [29, 41]. Considering that this study was limited to a single-center design, the relationship among IOH, hemoglobin levels, and blood loss requires further studies.

This study indicated that postoperative systolic blood pressure was an independent risk factor for IOH, which has not been previously reported [30, 42]. As the temperature decreases, blood vessels constrict, leading to an increase in blood pressure [43]. The effect of lower temperatures indicated a strong and acute effect of temperature on systolic blood. Decreases in temperature activate the sympathetic nervous system, leading to an increase in heart rate and the consequent risk of systolic blood pressure. A systematic review and meta-analysis found an inverse association between temperature and systolic blood pressure [44]. Patients with cardiovascular diseases may be more susceptible to temperature changes. Thus, improving IOH is required to manage and prevent cold-induced increases in systolic blood pressure. To date, the mechanisms underlying the relationship between temperature and blood pressure are not fully understood, and further studies are required.

## Limitations

Compared with similar studies, the following limitations should be highlighted in this study. First, all the risk factors affecting IOH were included in the literature review; therefore, additional risk factors must be explored. Second, the sample size was small, and it was just a single-center study; multicenter, large sample data are required to further validate the prediction model. Third, this study revealed that patients with high postoperative systolic blood pressure were more susceptible to IOH, which differs from the findings of previous studies and requires further verification. Therefore, more well-designed studies are required to strengthen the predictive performance of risk models.

## Conclusion

The risk prediction model has good predictive value and can accurately predict the occurrence of IOH in patients who undergo THA, which provides reliable guidance for clinical work and has good clinical application value.

## Data Availability

The data that support the findings of this study are available from the corresponding author, upon reasonable request.
